# Comparison of laser and circumlimbal suture induced elevation of intraocular pressure in albino CD-1 mice

**DOI:** 10.1371/journal.pone.0189094

**Published:** 2017-11-30

**Authors:** Hsin-Hua Liu, Liwei Zhang, Meng Shi, Lu Chen, John G. Flanagan

**Affiliations:** 1 School of Optometry and Vision Science, University of California, Berkeley, California, United States of America; 2 Center for Eye Disease and Development, Vision Science Graduate Program, University of California, Berkeley, California, United States of America; Bascom Palmer Eye Institute, UNITED STATES

## Abstract

Animal models of ocular hypertension are important tools for glaucoma studies. Both acute transient models and chronic models of ocular hypertension may be useful to investigate specific aspects of neurodegeneration. In this study, we compare the intraocular pressure (IOP) and inner retinal changes induced by 1) laser photocoagulation of both episcleral veins and limbal vessels and 2) circumlimbal suture in CD-1 mice. The suture group is divided into 3 subgroups depending on the level of the immediate IOP spike (acute > 55 mmHg or chronic < 55 mmHg) and time period of monitoring (7 or 28 days). The laser group is followed for 7 days. IOP data show that it peaks at 5 hours and returns to normal level within 7 days in the laser group. In all suture groups, IOP spikes initially and decreases gradually, but it remains significantly elevated at 7 days. In 7 days, the acute suture model generates rapid loss of retinal nerve fiber layer (RNFL) and retinal ganglion cells (RGCs) when compared to the gradual loss by the chronic suture model, possibly due to retinal ischemia and reperfusion within the first few hours after treatment. The laser model falls between the acute suture and chronic suture models resulting in less RNFL and RGC loss than the acute suture model but significantly more loss than the chronic suture model. These results suggest that when using suture models of IOP elevation, it is critical to take the initial IOP spike into consideration and to choose between the acute and chronic models depending on respective research purposes.

## Introduction

Glaucoma, a neurodegenerative disease, is one of the leading causes of irreversible blindness [1]. Its hallmark features include retinal ganglion cell (RGC) degeneration and progressive loss of visual field. Ocular hypertension is a major risk factor of this disease and the focus of disease management is to lower intraocular pressure (IOP) [2, 3]. In glaucoma research, animal models of ocular hypertension are pivotal and provide useful platforms for understanding the disease pathogenesis and development of therapeutic strategies [4, 5]. Current established in vivo glaucoma models include 1) laser cauterization of the perilimbal region, 2) intracameral injection of foreign materials, 3) episcleral vein saline injection and 4) episcleral vein obstruction. These models induce ocular hypertension via various mechanisms to impede aqueous humor outflow [5]. Among various animal species, murine is the most popular because it is relatively inexpensive and has biological and genetic features highly similar to those of humans [6–8]. In terms of techniques to induce IOP elevation, each approach has its own advantages and limitations [4, 6]. Acute transient models of ocular hypertension are generally easier to produce and may be helpful for investigating aspects of acute glaucoma, such as neurodegeneration due to transient IOP elevation. Models of chronic ocular hypertension are more difficult to achieve but will permit the investigation of chronic glaucoma with different aspects of neurodegeneration after sustained IOP elevation. To better simulate an individual type of glaucoma for specific neurodegenerative studies, it is essential to differentiate between acute and chronic animal models of IOP elevation.

Animal models of ocular hypertension induced by laser photocoagulation of the aqueous outflow pathway had been developed and widely used in glaucoma research [9–13]. Previous studies using an IOP model in albino CD-1 mice by laser photocoagulation of both the episcleral veins and limbal vessels showed that significant axon degeneration in the myelinated optic nerve [14], reduction of retinal nerve fiber layer (RNFL) thickness and death of RGCs [15] were found by 7 days post-treatment. In these studies, elevated IOP also returned to baseline within 7 days. This duration of IOP elevation is relatively transient when compared to other mouse models of IOP elevation, such as the microbead occlusion model [16, 17] and hypertonic saline injection model [18]. Another important feature of these studies is the use of albino CD-1 mice. It was suggested that the albino mouse strain is more appropriate than the pigmented strain for the laser photocoagulation approach, possibly due to the lack of melanin to absorb the laser energy on the targeted tissue [14].

Recently, a novel technique to induce IOP elevation by circumlimbal suture had been documented in rodents [19–23]. This model applies the idea of oculopression to impede the aqueous outflow pathway [24, 25]. In our group, we developed this suture model of chronic ocular hypertension in C57BL/6 mice with an IOP threshold less than 55 mmHg [23]. A unique characteristic of this model is the possibility to induce an initial IOP spike close to 70 mmHg immediately after suture placement when the animal is still under systemic anesthesia. This may lead to acute retinal injury. It has been demonstrated that there is no significant decrease in retinal blood flow until IOP is above 70 mmHg in C57BL/6 mice [26]. In our suture model, the IOP spike may be underestimated as it is known that anesthesia decreases IOP [27] and IOP may further increase once animals recover from general anesthesia. In this study, we are interested in exploring the mice with an immediate IOP spike above 55 mmHg after suture, and we compare the results of mice with IOP spike less than this value. We have previously reported that mice exhibiting an IOP spike below 55 mmHg did not have significant acute effects [23].

Since albino CD-1 mice have been established in the laser model, in this study, we aims to use this strain to further explore the characteristics of the suture model in both acute and chronic types and compare the results with the laser model. The patterns of ocular hypertension, changes in the anterior chamber depth (ACD) and the rate of inner retinal (RNFL and RGC) loss are investigated. There are 4 experimental groups in this study. Besides the laser group (7 days), the animals with suture treatment are divided into 3 subgroups depending on the level of the IOP spike immediately following suture placement and the time period of monitoring (acute suture 7 days, IOP spike > 55 mmHg; chronic suture 7 days, IOP spike < 55 mmHg; chronic suture 28 days, IOP spike < 55 mmHg). We do not attempt to designate any animal prior to treatment. The IOP spike level post-treatment in each animal is used for grouping.

## Materials and methods

### Animals

All animals were treated in accordance with the ARVO Statement for the Use of Animals in Ophthalmic and Vision Research, and all procedures were approved by the Animal Care and Use Committee of University of California, Berkeley. Forty six-week old male CD-1 mice were purchased from the Charles River Laboratories (Wilmington, MA). Young adult animals were used in our study to avoid the confounding effect, ageing [26] and it is known that the structures of the iridocorneal angles of the mice mature by postnatal day 35 to 42 [28]. All animals were allowed to acclimatize to the housing facility for two weeks prior to experimentation. Food and water were available freely. Mice with complications due to technical failure (i.e. hyphema, corneal neovascularization), which occurred in 1 of 10 mice in the laser photocoagulation group and 9 of 40 mice in the suture groups, were excluded from the study.

### Laser photocoagulation

Animals were anesthetized with a mixture of ketamine/xylazine (100mg/kg and 10mg/kg, respectively, intraperitoneal) on a veterinary heating pad. A drop of topical anesthetic (proparacaine hydrochloride, 0.5%, Akorn, Lake Forest, IL) was applied to the cornea of a randomly selected eye. The laser procedure was performed as we recently published [15]. Briefly, animals were given monocular laser photocoagulation (532 nm, 300 mW x 0.5 seconds per spot, OcuLight TX, IRIDEX Corporation, Mountain View, CA) of the episcleral veins (70 ~ 80 spots) combined with 270° limbal vessels (sparing nasal 90°, 110 ~ 120 spots). After treatment, antibiotic ointment (Ak-Poly-Bac, Akorn, Lake Forest, IL) was added to the treated eye. The fellow untreated eye served as a within animal control.

### Circumlimbal suture

The procedure of circumlimbal suture implantation was detailed in our previous study [23]. In brief, animals were given the same systemic and local anesthesia as described in the laser photocoagulation procedure. A unilateral subconjunctival circumlimbal suture (10/0, nylon, Fine Science Tools, Foster City, CA) was placed around the equator (0.4 ~ 0.5 mm behind the limbus) of a randomly selected eye by 5 anchor points and double knots on the conjunctiva. To induce an immediate IOP spike among sutured eyes, the tightness of each ending knot was controlled by pulling it until the suture was closed to form a knot. The suture threaded through subconjunctiva (in and out) and was placed underneath major episcleral veins to avoid direct suture compression on those vessels. To reduce irritation, the residual suture on the knots was cut. The contralateral control eye was untreated and served as a within animal control. Antibiotic ointment was added to the eye following treatment. The suture was left in place for either 7 days or 28 days depending on the group design.

### IOP measurement

Measurements of IOP were carried out in conscious animals with a Tonolab tonometer (iCare, Helsinki, Finland) except for the reading measured immediately after treatment, as we previously published [23]. Baseline IOP was monitored one day prior to IOP induction. IOP was measured immediately, 3, 5, 8, 10, 12 and 24 hours after treatment, daily for the next 6 days in all 7-day groups. For the 28-day suture group, it was measured immediately, 3 and 24 hours after treatment and every 3 days for the rest experimental period. To control for diurnal fluctuation, IOP was measured between 10 am ~ 12 pm under identical lighting conditions. Average of 10 repeated readings was recorded to return a single IOP data.

### Optical coherence tomography

The analysis was performed as we previously published [23]. Following anesthesia, animals were placed on the imaging platform for OCT measurement. A Bioptigen spectral domain OCT system (Durham, NC) was used to capture anterior chamber and retinal images. A 3 mm x 3 mm rectangular (both 0° and 90°) scanning sequence produced a single *en-face* image using a customized mouse lens (50° field of view), which consisted of 100 B-scan images, with every B-scan comprising 1000 A-scans. The anterior chamber imaging was performed first without pupil dilation. The center of the pupil was centralized in the *en-face* image to allow analysis of the central ACD. For retinal imaging, the pupil was dilated with a drop of tropicamide (0.5%, Akorn, Lake Forest, IL) and phenylephrine (2.5%, Paragon BioTeck, Portland, OR). To enable B-scan image analysis at the optic nerve head, it was also centralized in the *en-face* image. Lubricant eye drops (Systane Ultra, Alcon, Fort Worth, TX) were used during imaging to maintain corneal moisture. The caliper function in the InVivoVue Clinic software was used to analyze the B-scan images. The ACD was measured from the center of the pupil on the lens vault to the posterior aspect of the central cornea (S1A Fig). The total retinal thickness was measured from the RNFL to retinal pigment epithelium (RPE) with both layers included (S1B Fig). The retinal layer thickness was measured in 4 quadrants (nasal, temporal, superior and inferior) of the *en-face* image. Three measurements within each quadrant were performed (400, 500 and 600 μm from the center of the optic nerve head, S1B Fig). A total of 12 readings were averaged to return one single thickness data. All OCT measurements were conducted by masked observers.

### Retinal whole-mount and RGC quantification

The procedure was performed as we previously published [23]. Briefly, eyeballs were enucleated, fixed with 4% paraformaldehyde and washed in phosphate-buffered saline (PBS). The retina was rinsed in PBS and flattened by four radial cuts. After fixation with methanol to allow permeabilization, the retina was rinsed with PBS and incubated with a goat-anti-mouse primary antibody against Brn3a (brain-specific homeobox/POU domain protein 3A, Santa Cruz Biotechnology, Santa Cruz, CA), which was visualized with a secondary antibody, Alexa Fluor donkey anti-goat IgG (Jackson ImmunoResearch Laboratories, West Grove, PA). Samples were mounted using an antifade mounting medium (Prolong Gold, Invitrogen, Carlsbad, CA) and imaged with a Zeiss Axioplan 2 Imaging epifluorescent microscope system (Carl Zeiss, Oberkochen, Germany). Two areas equal in size (450 x 320 μm, 20x magnification, 850 μm from the center of the optic nerve head) in each quadrant of the retina were imaged. The cell number in each area was quantified using the ImageJ software (National Institutes of Health, Bethesda, MD) in a masked manner. The cell number from all areas was averaged to produce a single value for each retina.

### Statistics

Data statistical analysis was executed with Prism 6 software (GraphPad, La Jolla, CA). Data were expressed as mean ± SD (standard deviation). For IOP, OCT and cell density data, paired t-test, one-way ANOVA and repeated measures (RM) two-way ANOVA with a Tukey’s or Bonferroni’s post-hoc test were used, where applicable. Relationships between changes in RNFL thickness and IOP peak / integral were evaluated by linear regression to establish statistical significance, with the strength of the relationship (goodness of fit) determined with a Pearson coefficient.

## Results

**Fig 1:**
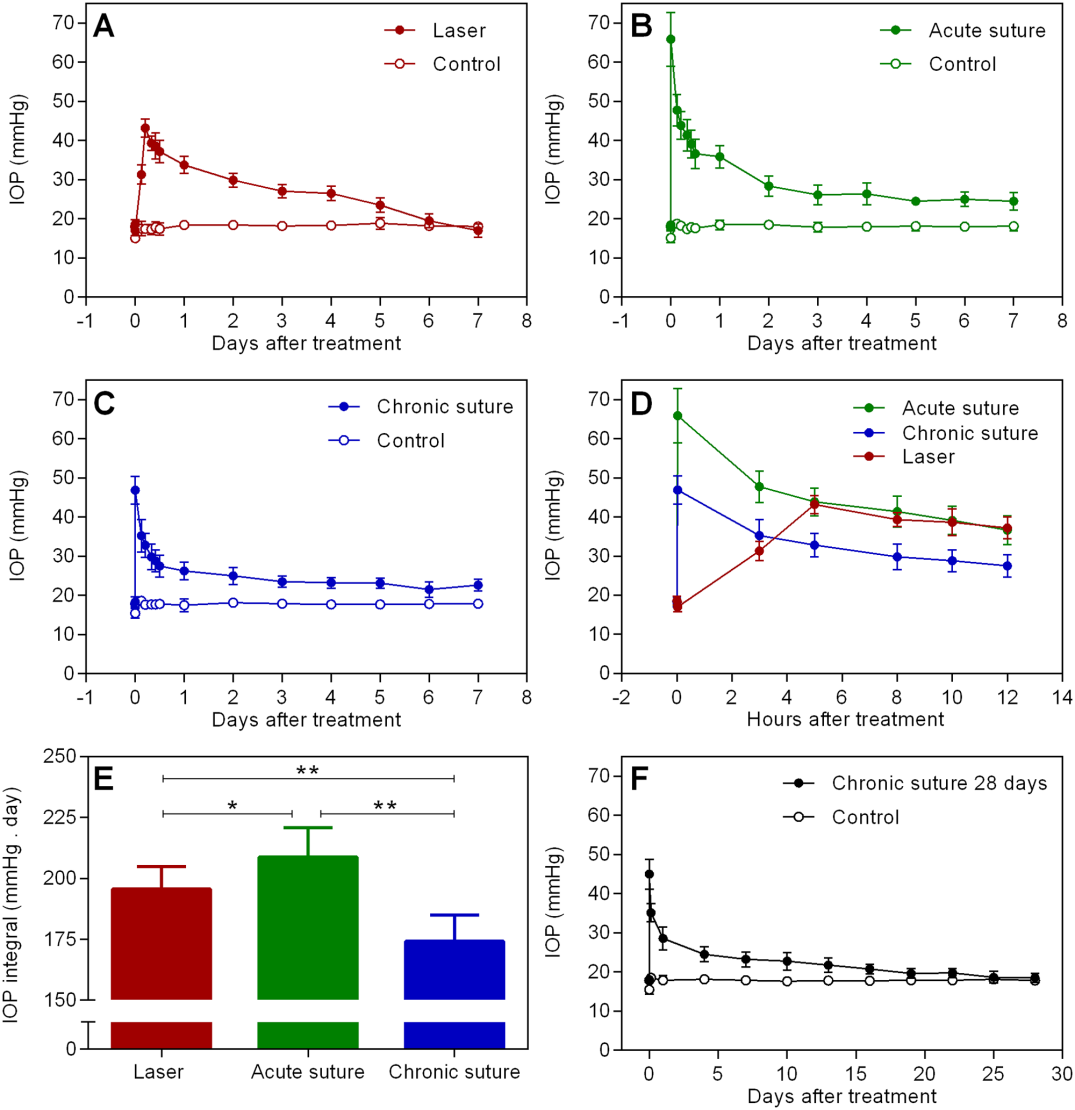
IOP data. Average IOP in the laser (A), acute suture (B) and chronic suture (C) group across 7 days. (D) Comparison of IOP in the treated eyes (first 12 hours) shown in Panels A, B and C. (E) Results of IOP integral over 7 days. The acute suture group had the highest IOP integral among the 3 groups, and IOP integral in the laser group was significantly greater than that in the chronic suture group. (F) Average IOP in the chronic suture 28-day group. * p < 0.05, ** p < 0.01, mean ± SD, n = 9 in the laser group, n = 8 in the acute suture group, n = 11 in the chronic suture group, n = 12 in the chronic suture 28-day group.

Fig 1 shows the IOP data across time in all groups (also see Table 1 for IOP data at specific time points). In the laser group (Panel A, n = 9), there was no immediate IOP spike following treatment (17.0 ± 1.2 mmHg). IOP elevated to 43.2 ± 2.3 mmHg after 5 hours and it gradually reduced afterwards. At day 7, it reduced to normal when compared to contralateral control eyes (17.0 ± 1.7 vs 18.1 ± 1.1 mmHg, p > 0.99, RM two-way ANOVA with a Bonferroni’s post-hoc analysis). In the acute suture group (Panel B, n = 8), the immediate IOP spike was 65.9 ± 6.9 mmHg, ranging from 57 to 76 mmHg. In contrast to the laser group, IOP remained elevated at day 7 (24.5 ± 2.2 vs control eyes 18.1 ± 1.1 mmHg, p < 0.01, RM two-way ANOVA with a Bonferroni’s post-hoc analysis). In the chronic suture 7-day group (Panel C, n = 11), the IOP profile was similar to that in the acute suture group except for a lower IOP spike (46.9 ± 3.5 mmHg, ranging from 41 to 52 mmHg). Panel D compares the IOP data (first 12 hours) of treated eyes in the 3 groups. It is worth mentioning that, at 5 hours, IOP in the laser group (43.2 ± 2.3 mmHg) was similar to that in the acute suture group (43.9 ± 3.5 mmHg, p = 0.91, RM two-way ANOVA with a Tukey’s post-hoc test) and significantly higher than that in the chronic suture group (32.8 ± 3.0 mmHg, p < 0.01). IOP integral over 7 days for the 3 groups is shown in Panel E. The laser group had significantly less IOP integral (196 ± 9 mmHg.day) than the acute suture group (209 ± 12 mmHg.day, p = 0.047, one-way ANOVA with a Tukey’s post-hoc test) and had significantly greater IOP integral than the chronic suture group (174 ± 11 mmHg.day, p < 0.01). The chronic suture 28-day group (Panel F, n = 12) had a similar IOP pattern when compared to the chronic suture 7-day group. At day 28, IOP was 18.5 ± 1.2 mmHg in the treated eyes and 17.8 ± 0.8 mmHg in the control eyes (p > 0.99, RM two-way ANOVA with a Bonferroni’s post-hoc analysis).

**Table 1:** Comparison of group details and IOP data at specific time points.

Group	Laser	Acute suture	Chronic suture	Chronic suture
Period (days)	7	7	7	28
IOP baseline (mmHg)	18.7 ± 1.1	18.5 ± 1.1	18.4 ± 1.3	18.1 ± 1.0
IOP immediate after treatment (mmHg)	17.0 ± 1.2	65.9 ± 6.9	46.9 ± 3.5	45.0 ± 3.8
IOP at 5 hours after treatment (mmHg)	43.2 ± 2.3	43.9 ± 3.5	32.8 ± 3.0	n/a
IOP at the end of experiment (mmHg)	17.0 ± 1.7	24.5 ± 2.2	22.6 ± 1.5	18.5 ± 1.2

Mean ± SD, n = 8 ~12.

**Fig 2:**
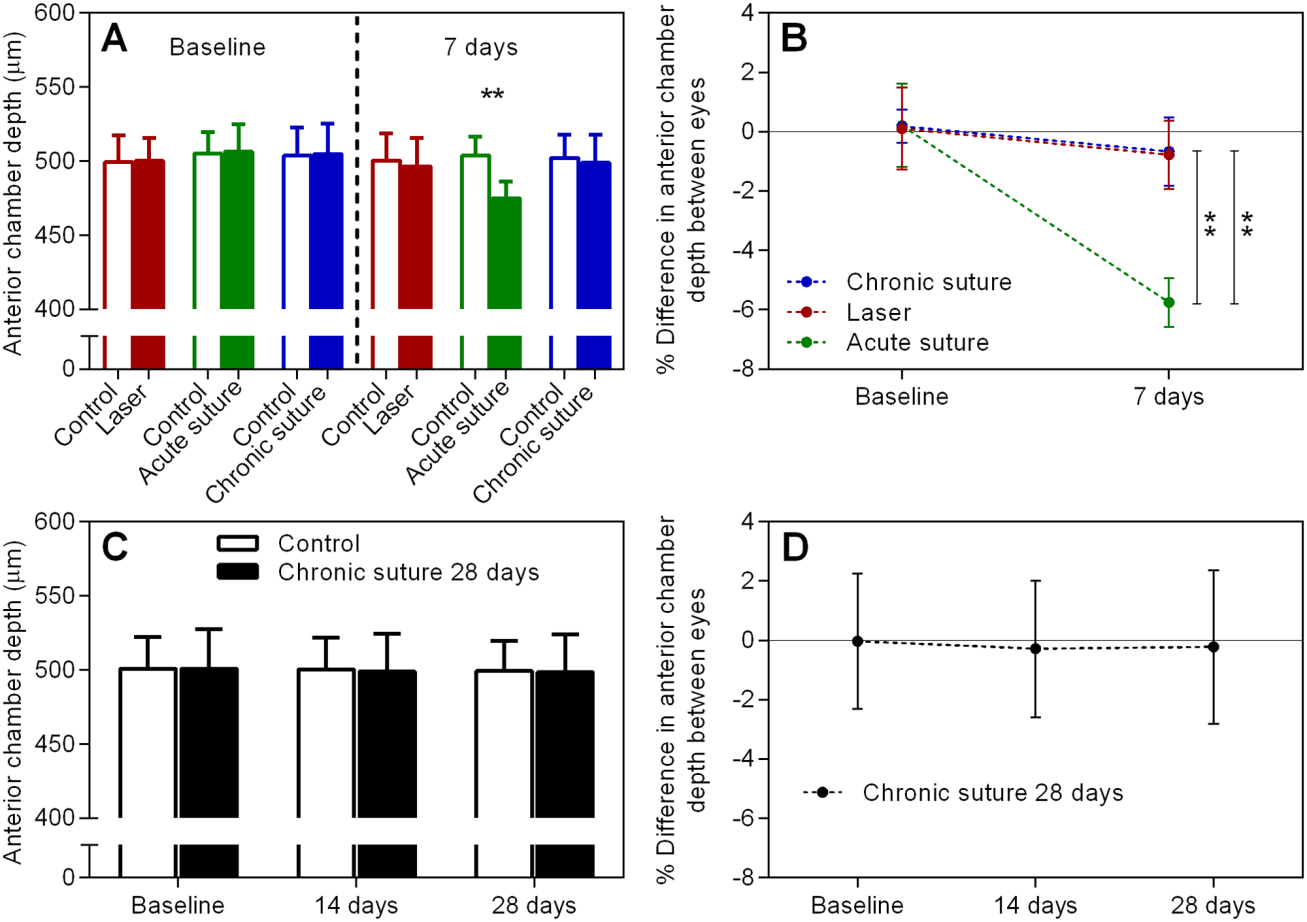
ACD data. (A) Average ACD at baseline and 7 days in all 7-day groups. (B) The percentage change between treated and control eyes in the groups shown in (A). (C) & (D) as described for (A) & (B), individually, but for the chronic suture 28-day group. ** p < 0.01, mean ± SD, n = 9 in the laser group, n = 8 in the acute suture group, n = 11 in the chronic suture group, n = 12 in the chronic suture 28-day group.

The ACD data measured by the OCT is shown in Fig 2. The ACD at baseline and day 7 is shown in Panel A. The acute suture group had significantly reduced ACD at day 7 (p < 0.01, paired t-test). Panel B shows the percentage change in ACD between treated and control eyes ((treated—control)/control). At day 7, the acute suture group had a significantly greater reduction in ACD (-5.8 ± 0.8%) than the laser group (-0.8 ± 1.2%, p < 0.01, RM two-way ANOVA with a Tukey’s post-hoc test) and the chronic suture group (-0.7 ± 1.2%, p < 0.01). No significant difference was found between the laser and chronic suture group (p = 0.97). Panels C & D show the ACD and percentage change in the chronic suture 28-day group, respectively. The relative change was -0.3 ± 2.3% at 14 days and -0.2 ± 2.6% at 28 days.

**Fig 3:**
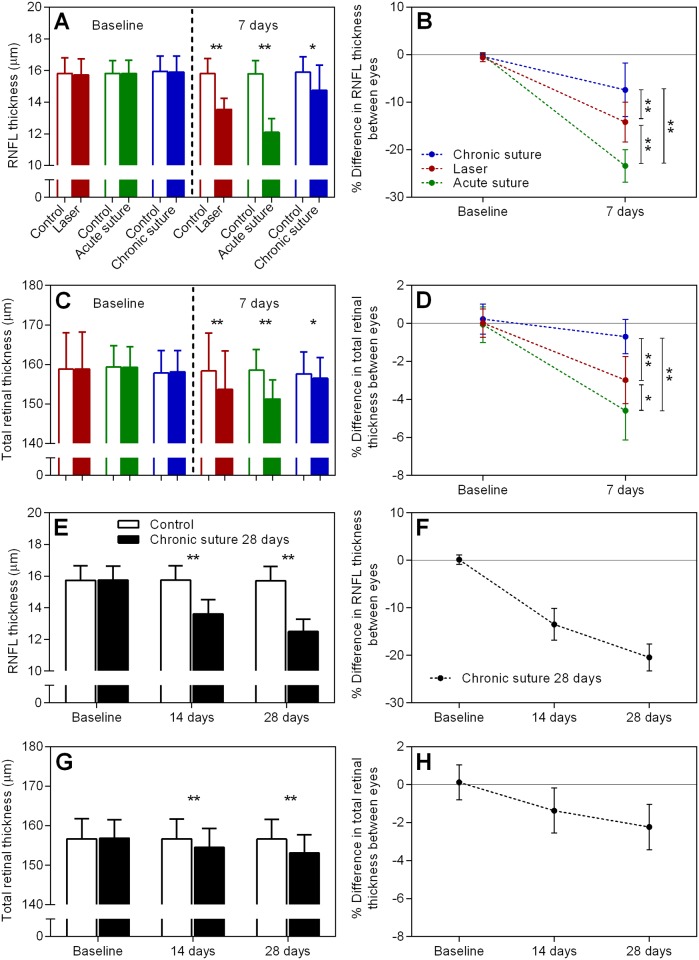
Retinal layer thickness results. (A) Average RNFL thickness at baseline and day 7 in all 7-day groups. (B) The relative change in RNFL thickness in all 7-day groups. (C) & (D) as described in (A) and (B), individually, but for average total retinal thickness. (E) Average RNFL thickness at baseline, day 14 and day 28 in the chronic suture 28-day group. (F) The relative difference in RNFL thickness across 28 days. (G) & (H) as described in (E) and (F), respectively, but for average total retinal thickness. * p < 0.05, ** p < 0.01, mean ± SD, n = 9 in the laser group, n = 8 in the acute suture group, n = 11 in the chronic suture group, n = 12 in the chronic suture 28-day group.

Fig 3 demonstrates the data of RNFL and total retinal thickness evaluated by the OCT. RNFL thickness was significantly decreased in all groups at day 7 (p < 0.05, paired t-test, Panel A). At day 7, the laser group had a relative reduction of -14.2 ± 4.2% (Panel B), significantly less than the acute suture group (-23.4 ± 3.4%, p < 0.01, RM two-way ANOVA with a Tukey’s post-hoc test) and significantly greater than the chronic suture group (-7.4 ± 5.6%, p < 0.01). Panels C and D reveal the total retinal thickness and relative change, respectively. A similar result was observed for total retinal thickness. At day 7, the relative reduction was -4.6 ± 1.5% in the acute suture group, -3.0 ± 1.2% in the laser group and -0.7 ± 0.9% in the chronic suture group. Panels E & F show the RNFL thickness and relative change in the chronic suture 28-day group. A progressive loss of RNFL was found in this group (-13.5 ± 3.3% at 14 days; -20.4 ± 2.8% at 28 days). The total retinal thickness in this group is shown in Panel G and relative change shown in Panel H. A similar trend of loss in total retinal thickness was noted when compared to the RNFL loss (-1.4 ± 1.2% at 14 days; -2.2 ± 1.2% at 28 days).

**Fig 4:**
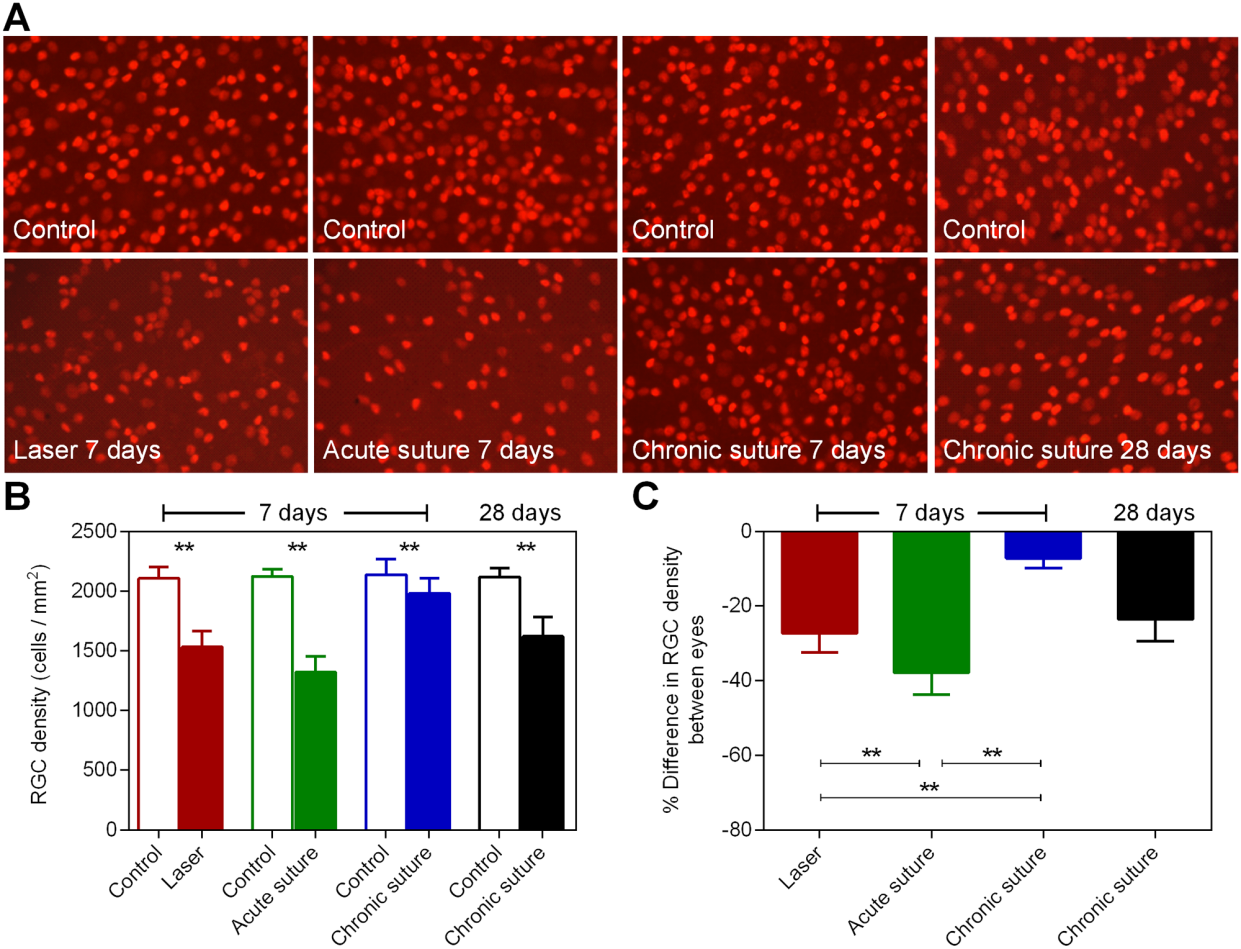
RGC density results. (A) Representative retinal whole-mount images (450 x 320 μm, 850 μm from the center of the optic nerve head, 20x magnification) with Brn3a-labeled RGCs from both eyes in all groups. (B) Average RGC density in all groups. (C) The relative difference in RGC density between eyes for all groups. ** p < 0.01, mean ± SD, n = 9 in the laser group, n = 8 in the acute suture group, n = 11 in the chronic suture group, n = 12 in the chronic suture 28-day group.

At the end of experiment following OCT measurement, retinal samples were harvested for RGC density assessment (Fig 4). The representative retinal section (450 x 320 μm) with labeled RGCs for each group is shown in Panel A. The cell density in all groups is shown in Panel B. All groups had significantly reduced cell density. The relative change in RGC density for the laser group was -27.3 ± 5.1% (Panel C), significantly less than that in the acute suture group (-37.8 ± 5.9%, p < 0.01, one-way ANOVA with a Tukey’s post-hoc test) and significantly greater than that in the chronic suture group (-7.2 ± 2.6%, p < 0.01). In the chronic suture 28-day group, the loss of RGC density was -23.5 ± 5.9%, implying that the loss was progressive.

**Fig 5:**
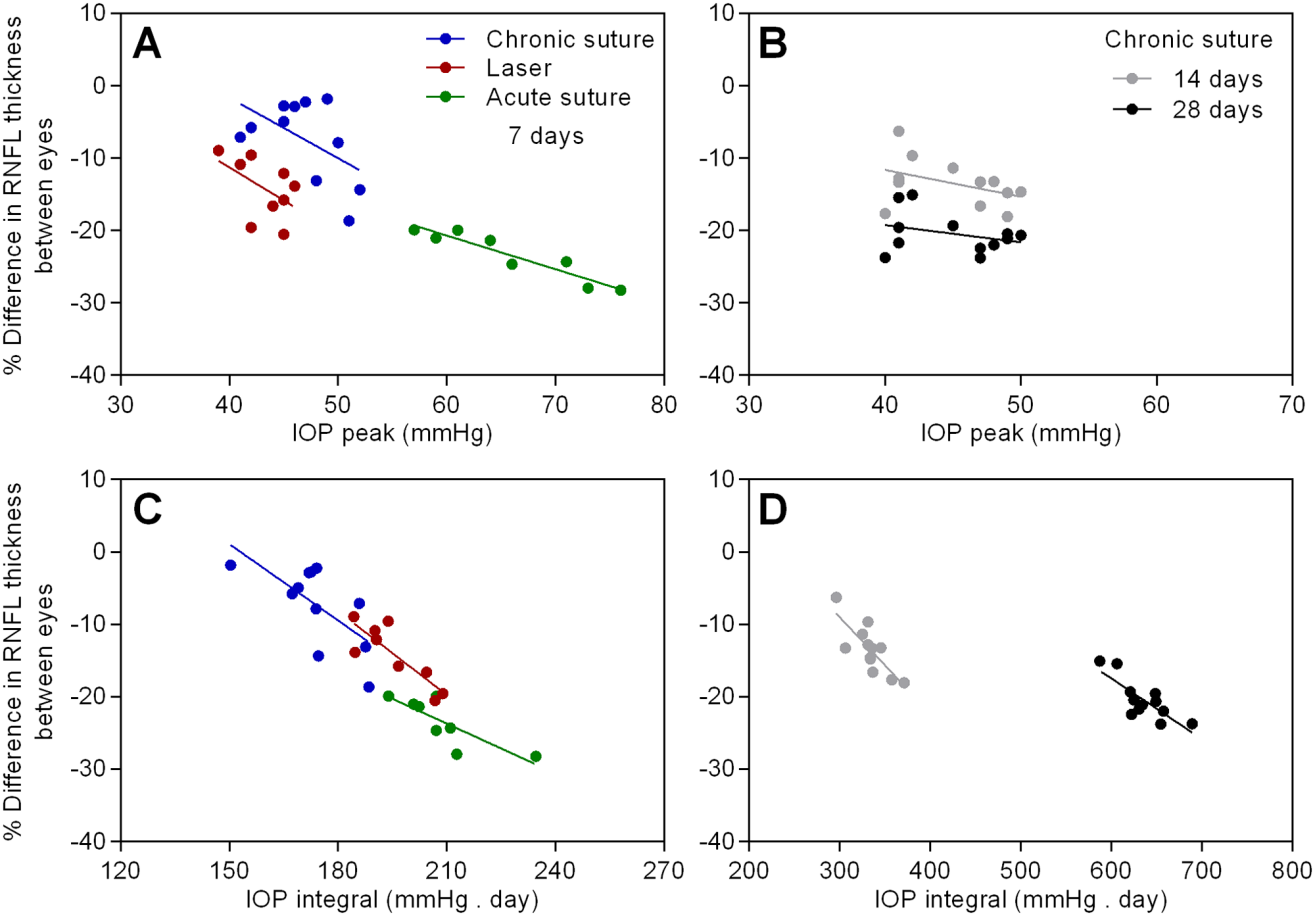
The linear correlation between the relative change in RNFL thickness and IOP peak / integral in all groups. n = 9 in the laser group, n = 8 in the acute suture group, n = 11 in the chronic suture group, n = 12 in the chronic suture 28-day group.

Fig 5 shows the linear correlation between the relative change in RNFL thickness and IOP peak / integral in all groups. We referred “IOP peak” to the maximal IOP recorded during the entire experimental period. It was at 5 hours for the laser group and immediate after suture placement for all suture groups. In Panel A, no significant correlation between IOP peak and the relative loss of RNFL was found for the chronic suture group (r^2^ = 0.28, p = 0.09) and the laser group (r^2^ = 0.26, p = 0.16). A strong correlation was found for the acute suture group (r^2^ = 0.89, p < 0.001). For the chronic suture 28-day group (Panel B), no correlation was found at 14 days (r^2^ = 0.17, p = 0.18) and 28 days (r^2^ = 0.10, p = 0.31). Panel C shows the correlation with IOP integral. All groups showed a significant correlation (the chronic suture group: r^2^ = 0.46, p = 0.02; the laser group: r^2^ = 0.72, p < 0.01; the acute suture group: r^2^ = 0.66, p = 0.01). For the chronic suture 28-day group (Panel D), the correlation was significant at both 14 days (r^2^ = 0.63, p < 0.01) and 28 days (r^2^ = 0.64, p < 0.01).

## Discussion

In the current study, we demonstrated that IOP elevation was relatively transient in the laser group when compared to all suture groups. The acute suture group had the greatest IOP integral over 7 days, and the chronic suture group had the least. In addition, the anterior chamber became significantly shallower in the acute suture group post-treatment. ACD was similar between the laser group and the chronic suture group. All groups had significant thinning of RNFL at day 7, with the acute suture group exhibiting the greatest loss and the chronic suture group giving the least. The trend of Brn3a+ RGC loss was similar to the thinning of RNFL. A significant correlation between IOP peak and the loss of RNFL was noted in the acute suture group only. In the chronic suture 28-day group, there was gradual loss of RNFL and Brn3a+ RGCs. The loss of RNFL was significantly correlated with IOP integral only.

The IOP profile in the laser group was comparable with previous studies utilizing similar laser treatment [13–15]. For the suture groups, 8 of total 31 animals (26%) exhibited an initial IOP spike > 55 mmHg (acute suture group). This ratio is similar to that reported in our previous study [23]. Although we attempted to obtain identical suture tightness for each suture placement, certain animals had a relatively higher IOP spike, possibly because of individual disparity. In the clinic, ACD is an important indicator to estimate the risk of angle closure glaucoma [29]. When the ACD decreases, the risk increases. Our OCT results revealed the shallowest ACD in the acute suture group (Fig 2B). This finding implies that the acute suture group may have the greatest risk of angle closure leading to high IOP. It is not surprising to observe a greater IOP integral in the acute suture group, as IOP was consistently higher than that in the other two groups, at least for the first 12 hours after treatment (Fig 1D). Despite normalization of IOP within 7 days and absence of an initial IOP spike observed in the laser group, the IOP integral was larger than that in the chronic suture group. Indeed, from 5 to 12 hours post-treatment, IOP was higher in the laser group. IOP in the chronic suture group reduced rapidly in this time period following the initial spike, presenting a different pattern when compared to the laser group, where IOP elevated gradually and peaked around 5 hours after treatment. An advantage of the suture model is that it is possible to adjust the tightness of the suture to achieve a range of IOP levels. It is also possible to induce IOP reduction by suture removal, as we demonstrated previously [21, 23]. Another advantage of both the laser and suture models over other established models is that it does not require multiple interventions as seen in the microbead injection model [30] and it is less difficult to perform than the hypertonic saline model [5].

The relatively severe retinal injury seen in the acute suture group may be associated with retinal ischemia triggered by the extreme IOP spike within the first few hours post-treatment. Given the suture model was minimally invasive and the ocular immune privilege was relatively preserved [19], it seems unlikely that the observed retinal injury is caused by inflammation-related responses. In the acute suture group, the average immediate IOP spike was 65.9 mmHg measured at 3 ~ 5 minutes after suture placement and 20 ~ 30 minutes following systemic anesthesia. It had been reported that in mice with normal awake IOP, the IOPs were reduced by ~48%, 30 minutes after anesthesia using ketamine+xylazine [31]. Another study demonstrated that ketamine+xylazine injected intraperitoneally could induce a duration of immobilization for ~70 minutes and a duration of surgical anesthesia (loss of pedal withdrawal reflex) for ~30 minutes in mice [32]. As the general anesthesia produces a profound hypotensive effect [31, 33], it is likely that these animals have been exposed to an even higher IOP insult close to 70 mmHg, a threshold for significant retinal blood flow reduction in mice [26]. The duration of retinal ischemia might be transient and reperfusion occurred subsequently since IOP recovered to 47.8 ± 4.1 mmHg at 3 hours and 35.9 ± 2.9 mmHg at day 1. However, this speculation needs further investigation. In previous rat studies of retinal ischemia and reperfusion, disruption of mitochondria, degeneration of axons, disordered myelin sheaths and alterations of extracellular matrix in the optic nerve were found [34, 35]. Not surprisingly, retinal ischemia is likely to cause pan-retinal damage in addition to loss of inner retinal neurons [36–39]. In our retinal OCT data of the acute suture group, the loss of RNFL in the treated eyes from baseline to day 7 was 3.7 ± 0.6 μm, whereas the total retinal loss was 8.0 ± 1.2 μm, implying that the thinning of retinal layers is not confined to the nerve fiber layer. In contrast, in the chronic suture group, the loss of RNFL was 1.1 ± 0.8 μm which could account for the majority of total retinal loss (1.6 ± 0.7 μm). These observations are in agreement with previous reports. As expected, there was a strong correlation between IOP peak and the loss of RNFL (Fig 5A), suggesting that the IOP spike plays a key role in the acute suture group. Also, the trend of RGC density loss among the three 7-day groups (Fig 4C) is similar to that for the RNFL thinning (Fig 3B).

It is surprising that the chronic suture group demonstrated significant inner retinal damage at 7 days, although the degree was mild. However, the finding that progressive injuries in the inner retina from 14 to 28 days were significantly correlated with IOP integral rather than IOP peak is consistent with our previous report [23]. An interesting finding in this study is that, in albino CD-1 mice, the relative loss of RNFL at 14 and 28 days after suture placement was -13.5 ± 3.3% and -20.4 ± 2.8%, respectively; however, in C57BL/6 mice, it was -0.3 ± 2.3% and -4.0 ± 3.8% [23]. The development of inner retinal injury induced by the chronic suture model appears to be more rapid in albino CD-1 mice than in pigmented C57BL/6 mice at the same age. A similar strain-specific susceptibility to inner retinal injury induced by experimental ocular hypertension was also reported in previous studies using the microbead injection model [40]. Understanding the underlying mechanism for this disparity may provide an important insight into risk factors of glaucoma. It is possible that the difference in biomechanical properties of the sclera between mouse strains may account for the different susceptibility [41, 42]. However, this speculation requires further investigation. For the laser group, although IOP elevation only lasted for less than a week, it is sufficient to induce a higher magnitude of inner retinal damage than the chronic suture group. This may be due to a significantly greater IOP integral in the laser group.

In conclusion, relatively rapid loss in RNFL and RGCs is observed in the acute suture group and is significantly correlated with the initial high IOP spike. The chronic suture group yields progressive inner retinal loss associated with IOP integral over time. The laser model also appears to be more acute in nature, generating rapid loss of RNFL and RGCs. When using suture models of ocular hypertension, particularly in mice, it is important to evaluate the initial IOP spike and to distinguish between the acute and chronic models for respective research purposes. For the acute suture model, it may be useful for acute closure glaucoma studies because a significantly shallower ACD and acute inner retinal damage were found. The chronic suture model may be used for open angle glaucoma studies as it largely mimics its characteristics. Further investigation using this repertoire of mouse models may provide new insights into the mechanisms and management of ocular hypertension and glaucoma of different types and at various stages.

## Supporting information

S1 Fig (A) Anterior chamber depth (the length of the double head arrow) was measured between the central pupil on the lens vault and the posterior aspect of the central cornea. (B) The RNFL and total retinal thickness were measured at 400, 500 and 600 μm from the center of the optic nerve head in each quadrant. Three measurements in all 4 quadrants (nasal, temporal, superior and inferior) of the retina were averaged to yield a single reading.
(TIF)
