# Supplementary figures and images for: Comparison of laser and circumlimbal suture induced elevation of intraocular pressure in albino CD-1 mice
Source: PLoS One. 2017 Nov 30;12(11):e0189094. doi: 10.1371/journal.pone.0189094 (PMC5708743; doi:10.1371/journal.pone.0189094)

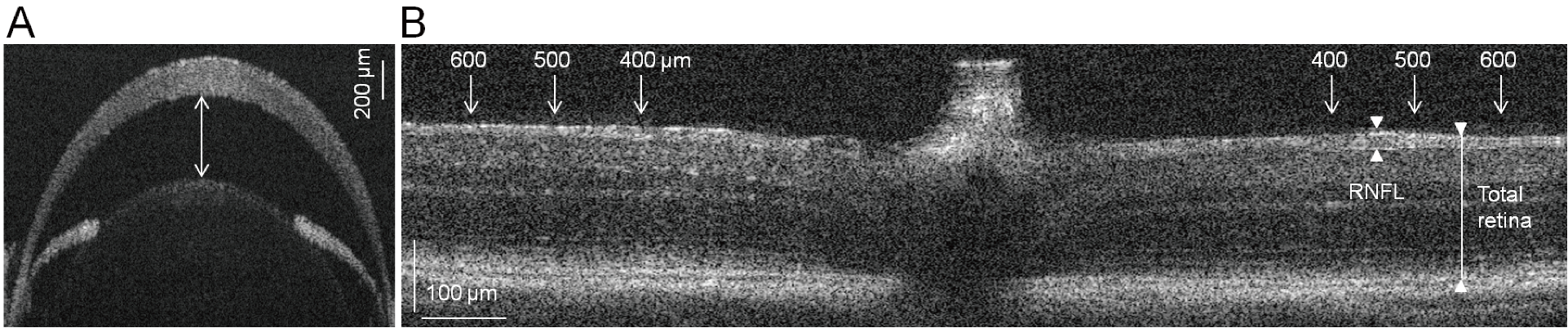

Supplement: S1 Fig — (A) Anterior chamber depth (the length of the double head arrow) was measured between the central pupil on the lens vault and the posterior aspect of the central cornea. (B) The RNFL and total retinal thickness were measured at 400, 500 and 600 μm from the center of the optic nerve head in each quadrant. Three measurements in all 4 quadrants (nasal, temporal, superior and inferior) of the retina were averaged to yield a single reading. (TIF) [file pone.0189094.s001.tif]
